# Street Food in Maputo, Mozambique: The Coexistence of Minimally Processed and Ultra-Processed Foods in a Country under Nutrition Transition

**DOI:** 10.3390/foods10112561

**Published:** 2021-10-23

**Authors:** Sofia Sousa, Marcello Gelormini, Albertino Damasceno, Simão A. Lopes, Sérgio Maló, Célia Chongole, Paulino Muholove, Pedro Moreira, Nuno Lunet, Patrícia Padrão

**Affiliations:** 1 EPIUnit—Instituto de Saúde Pública, Universidade do Porto, Rua das Taipas 135, 4050-600 Porto, Portugal; sofia.sousa@ispup.up.pt (S.S.); tino_7117@hotmail.com (A.D.); pedromoreira@fcna.up.pt (P.M.); nlunet@med.up.pt (N.L.); 2 Laboratório para a Investigação Integrativa e Translacional em Saúde Populacional (ITR), Rua das Taipas 135, 4050-600 Porto, Portugal; 3 Faculdade de Ciências da Nutrição e Alimentação, Universidade do Porto, Rua do Campo Alegre 823, 4150-180 Porto, Portugal; 4 Agência Italiana para a Cooperação e Desenvolvimento, Rua Damião de Góis 381, 1100 Maputo, Mozambique; marcello.gelormini@gmail.com; 5 Faculdade de Medicina, Universidade Eduardo Mondlane, Avenida Salvador Allende 702, 1100 Maputo, Mozambique; 6 Faculdade de Medicina, Universidade do Porto, Alameda Prof. Hernâni Monteiro, 4200-319 Porto, Portugal; 7 Departamento de Matemática e Informática, Universidade Eduardo Mondlane, Av. Julius Nyerere 3453, 1100 Maputo, Mozambique; simaoantonio612@gmail.com; 8 Departamento de Geografia, Universidade Eduardo Mondlane, Av. Julius Nyerere 3453, 1100 Maputo, Mozambique; malo_gis@yahoo.com (S.M.); cchongole@gmail.com (C.C.); muholove@gmail.com (P.M.); 9 Centro de Investigação em Atividade Física, Saúde e Lazer, Faculdade de Desporto, Universidade do Porto, Rua Dr. Plácido Costa 91, 4200-450 Porto, Portugal

**Keywords:** street food, nutritional value, processing degree, nutrition transition, Africa

## Abstract

The aim was to characterise the extent of processing and nutritional composition of the street foods offered in Maputo, Mozambique. A cross-sectional study was conducted in October–November 2014 in the urban district of KaMpfumu. Twenty public transport stops were randomly selected, around which 500 meters buffers were drawn. All streets within these buffers were canvassed to identify all street food vending sites. Street food offer was assessed through interviews. Nutritional composition was estimated using standardised recipes (for homemade foods), food labels (for industrial products) and food composition tables (for *in natura* foods). The processing extent was classified using the “NOVA” food classification. A total of 810 vending sites were assessed. Unprocessed/minimally processed foods were available at 70.5% of vending sites (mainly fruit, water, and tea) and ultra-processed foods at 59.0% (mostly cakes, cookies, confectionery, and soft drinks). Energy content per 100 g of unprocessed or minimally processed foods was significantly lower than in all other food groups. In all food groups, contribution to total energy value was highest for carbohydrates (range: 33.1–51.2%), followed by fats (range: 29.3–36.0%) and protein (range: 6.8–18.6%). Public health policies targeting the improvement of this urban food environment should consider not only the nutritional composition but also the processing extent of the foods and beverages available.

## 1. Introduction

Nutrition transition can be defined as a set of “large shifts in diet and activity patterns, especially their structure and overall composition”, as a consequence of demographic and socioeconomic changes, namely urbanisation [1]. In urban areas of developing countries that are under nutrition transition, the intake of non-processed or minimally processed foods, which tend to be richer in vitamins, minerals, and fibre (e.g., fruit, vegetables, legumes, and whole grains), has been declining. On the other hand, the consumption of highly processed foods, which generally have higher energy and fat density, and are richer in saturated and trans fatty acids, sugars, and sodium, has been increasing [2,3,4,5]. These trends may have consequences on the nutritional and health status of the populations, contributing to an increase in the prevalence of overweight or obesity, as well as other diet-related non-communicable diseases (NCD) [1,4,6].

Results from a survey conducted in Mozambique under the Stepwise Approach to Chronic Disease Risk Factor Surveillance (STEPS) research project showed very low fruit and vegetables consumption [7]. A study conducted in 2012–2013 on a convenience sample of adults in Maputo also reported that ultra-processed food products, namely chicken-powdered stocks and sugar-sweetened beverages, were frequently used [8]. These findings suggest a shift towards a more westernised food consumption pattern in the country, which have been strongly associated with urbanisation and growing income [9], also having deleterious consequences to this population’s health. In Mozambique, total mortality by NCD was 22.8% in 2010, increasing to 28.5% in 2019. Total deaths attributable to cardiovascular diseases grew from 9.4% in 2010 to 12.0% in 2019, and the proportion of cancer deaths increased from 3.7% to 5.1% in the same time period [10]. Adult prevalence of overweight and obesity in Mozambique has also suffered a steep increase in recent years, ranging from 18.3% to 30.5% in women and from 11.7% to 18.2% in men, between 2005 and 2014–2015 [11]. On the other hand, the prevalence of anaemia among women of reproductive age was high and rising (51.0% in 2016), stunting in children under 5 years of age (although slowly declining) remained very frequent (42.3% in 2015) [12], and undernourishment affected 38% of total population in 2005–2007 [13], denoting the existence of a double burden of malnutrition.

Other significant aspects related with lifestyle changes have been associated with modifications in the dietary patterns of urban societies. Shifts in employment structure associated with urbanisation have resulted in a drastic reduction in the time dedicated to the preparation and cooking of homemade meals, and the consequent increase in the consumption of away-from-home food [14,15]. In low- and middle-income countries, street food constitutes an increasingly popular food option, mostly due to its low cost and convenience [16,17], contributing significantly to the daily dietary intakes of urban populations [18]. As such, urban street food environments are expected to reflect the nutrition transition process occurring in those settings [19]. Data from the African Food Security Urban Network [20], showed that, in Maputo, whereas the proportion of households using supermarkets as their primary source of food is small (with only 8% of the surveyed households using this source weekly), the proportion of households obtaining their foods from informal/street food markets on a weekly basis is very high (92%). However, some public health concerns have been raised regarding street food in Maputo, related to the unsatisfactory quality of some foods, both at the microbiological [21] and at the nutritional level [22].

The assessment of the processing extent of the foods available in urban settings under nutrition transition may contribute to defining future public health policies towards NCD prevention. However, such information is limited, and, to the best of our knowledge, no data are available for Mozambique. Therefore, the present research aims to characterise street food offered in Maputo, Mozambique, specifically with respect to the extent of processing and nutritional composition. For this purpose, we used the “NOVA” food classification, which is an internationally recommended methodology for classifying foods based on the extent and purpose of food processing [23,24].

## 2. Methods

A cross-sectional assessment of street food vending sites in Maputo, Mozambique, was conducted between October and November 2014, as described in a previously published protocol [25].

### 2.1. Street Food Vending Sites Selection

Street food was defined, according to the Food and Agriculture Organization and WHO, as “ready-to-eat foods and beverages prepared and/or sold by vendors and handlers especially in streets and other similar public places, for immediate consumption or consumption at a later stage without further processing or preparation” [26,27]. Venues selling street food, including facilities such as carts, trucks, stands, or a variety of improvised informal setups (e.g., shopping carts, trunks of cars, sides of vans, buckets, coolers, etc.), as well as “in-transit” vending sites, were considered eligible for this analysis. Food establishments within four permanent walls, permanent storefront businesses, vending sites selling exclusively non-food products or raw foods not ready-to-eat, sites operating in closed public spaces (e.g., markets) or organised entities (e.g., farmers’ markets, food fairs), as well as food stalls and carts that were part of permanent stores or licensed establishments, were excluded.

All eligible street food vending sites operating in a predefined area (as detailed below) were selected for this study.

### 2.2. Sampling Procedure

Maputo municipality is divided into seven districts. This study was conducted within the urban district of KaMpfumu, located in the southern region of the city, since this area was expected to present the highest concentration of street food vending sites among all districts in Maputo [28].

Using data from the Government of Mozambique on the public transport stops’ distribution in the KaMpfumu district [28], as well as other maps from the Maputo Municipal Council (Conselho Municipal Cidade de Maputo) [29], 134 public transport stops were identified, from which 20 were randomly selected. Within each stop selected, the study area was defined considering a 500 meters buffer around each selected stop. Areas falling outside the administrative borders of the municipal district were excluded.

### 2.3. Data Collection

Within the selected area, field researchers worked in pairs, moving through every street accessible to the public and identifying all eligible street food vending sites.

Interviews were performed during business days, between 9:00 and 16:00. A structured questionnaire was applied in Portuguese by trained interviewers to collect information on the food offered, namely the type of foods and beverages available for sale, as well as portions sizes. Vending sites were then classified into mobile (if they changed their position, continuously or not, throughout the day or week) or stationary (if they had a fixed location).

To ensure that street food vendors were not interviewed twice, the questionnaire began with a control question asking if the vendor had already been interviewed. Furthermore, each vendor received a sticker with the logo of the research project, this way indicating to other researchers that this street food vending site has already participated in the study.

### 2.4. Nutritional Composition Estimation

The nutritional composition of the foods and beverages identified was estimated using different strategies, depending on their nature. For homemade foods and beverages, standardised recipes were used to estimate their nutritional composition. For each culinary preparation, the ingredients and respective portions used were defined according to results from a study which assessed food intake and cooking practices in adults from Maputo, which also provided specific insights regarding traditional eating habits within the Mozambican context [8]. The recipes were then simulated by two Mozambican nutritionists, while the quantities of all ingredients used were measured. The conversion of those ingredients into nutrients was performed using the Food Processor Nutrition Analysis^®^ software, version 11.1 for Windows^®^ (ESHA Research, Salem, OR, USA). In the case of industrial food products, nutritional information was retrieved from Mozambican food labels whenever those were available, or using Portuguese food labels otherwise, in which case the selected products were as similar as possible as those sold in Mozambique. For in natura foods (e.g., fruit, cassava, nuts), the Mozambican food composition table [30] was used whenever possible. For foods whose nutritional data were not available, nutritional composition was estimated using the food composition tables from Brazil [31] and South Africa [32].

Estimated nutritional data included energy and macronutrients (total fat, carbohydrates and protein) contents, and were expressed both per serving and per 100 g. For each food, contributions of macronutrients to the total energy value (TEV) were computed by converting their contents (in grams) to energy values (in kcal), using the Atwater general factors (4 kcal/g for protein and carbohydrates, and 9 kcal/g for total fat), and then by dividing these by the total energy content.

### 2.5. Processing Extent Classification

Based on the “NOVA” food classification [24], foods and beverages were classified according to their processing extent into three groups: (1) unprocessed or minimally processed foods; (2) processed foods; and (3) ultra-processed foods. The first group included edible parts of plants (e.g., seeds, fruits, leaves, roots) or animals (e.g., muscle, eggs, milk, viscera), fungi, algae, and water, as well as unprocessed foods which are mildly altered by processes that include removal of inedible or unwanted parts and that do not involve the addition of other ingredients, such as salt, sugar, or fats (e.g., drying, milling, pasteurization, refrigeration, freezing, boiling, or packaging). Culinary preparations based on one or more unprocessed or minimally processed food items were also included in this group. The second group is essentially made up of industrial products resulting from the addition of processed culinary ingredients (e.g., salt, sugar, oil) to unprocessed or minimally processed foods, by using various preservation or cooking methods, such as fermentation, smoking, or canning (e.g., bread, canned fish, cheese). The third group is composed of food products resulting from industrial processing methods with no domestic equivalent, usually with five or more ingredients, including additives, such as dyes, artificial sweeteners, flavours, and/or emulsifiers.

### 2.6. Statistical Analysis

Statistical analysis was performed using the software Statistical Package for Social Sciences (SPSS^®^), version 22.0 for Windows^®^ (SPSS Inc., Chicago, IL, USA). Descriptive statistics were computed for street food availability, energy, and macronutrient contents. The Pearson’s chi-square test was used to compare stationary and mobile street food vending sites regarding the foods and beverages offered. The Mann–Whitney test was used to compare food groups regarding their energy and macronutrient content. Results were considered statistically significant when the critical level of significance (*p*) was less than 0.05.

## 3. Results

A total of 968 street food vending sites was identified within the selected area, of which 810 (83.7%) were available for interviews and thus were assessed.

Data on the availability of foods and beverages from mobile and stationary street food vending sites, according to processing degree, are presented in Table 1. Unprocessed or minimally processed foods (including culinary preparations using them as base) were the most commonly available, in 70.5% of the street food vending sites evaluated, especially fruit (35.7%) and water and tea (30.0%). Ultra-processed foods were available in 59.0% of the vending sites, mostly cakes and cookies (63.0%), confectionery (57.2%), and soft drinks (52.6%). Processed foods were available less often, and were mostly bread (68.1%), sandwiches (48.7%), and fermented alcoholic beverages (23.5%). A predominance of unprocessed or minimally processed foods was observed in mobile street food vending sites (86.1% vs. 65.8%, *p* < 0.001), while stationary street food vending sites sold predominantly ultra-processed foods (71.6% vs. 17.1%, *p* < 0.001). Processed foods were also more frequently available in stationary vending sites (16.5% vs. 8.6%, *p* = 0.004).

Table 2 shows the energy content and macronutrient distribution of the foods and beverages available, according to processing degree. A high variability in terms of energy content and macronutrient values was observed. Mean energy content per 100 g ranged from 156 kcal in unprocessed or minimally processed foods to 281 kcal in ultra-processed foods. Regarding macronutrients, carbohydrates were the major contributors to TEV in all food groups (ranging from 33.1% in processed foods to 51.2% in ultra-processed foods), followed by fats (ranging from 29.3% in processed foods to 36.0% in unprocessed or minimally processed foods). Proteins presented the lowest energy contribution in all food groups (ranging from 6.8% in ultra-processed foods to 18.6% in processed foods). Energy per serving was found to be the highest in nuts and culinary preparations based on nuts and leaves (e.g., *matapa*), whereas the most energy-dense foods (kcal/100 g) were industrial popcorn, cakes, and cookies. The highest contributions to TEV of carbohydrates were observed in soft drinks, the richest sources of protein were ham and culinary preparations based on goat, and the highest contributions of total fat were observed in cheese and canned fish.

**Figure 1:**
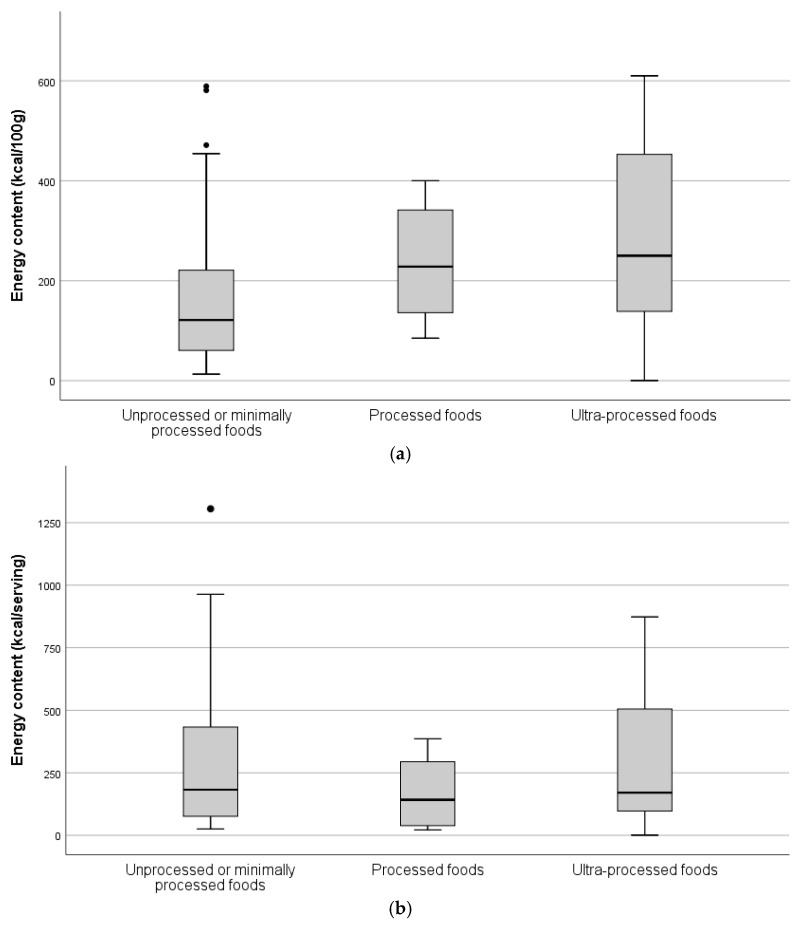
Estimated energy content per 100 g (**a**) and per serving (**b**) of the unprocessed/minimally processed, processed, and ultra-processed foods available (*n* = 810, vending sites available for interview).

**Figure 2:**
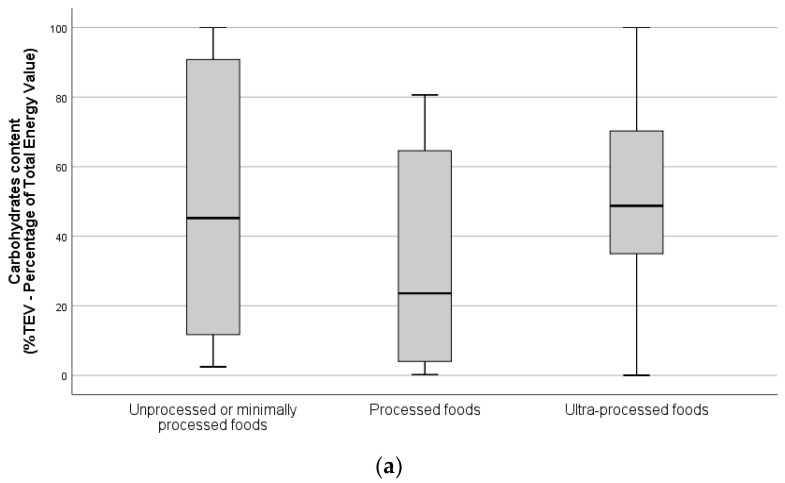

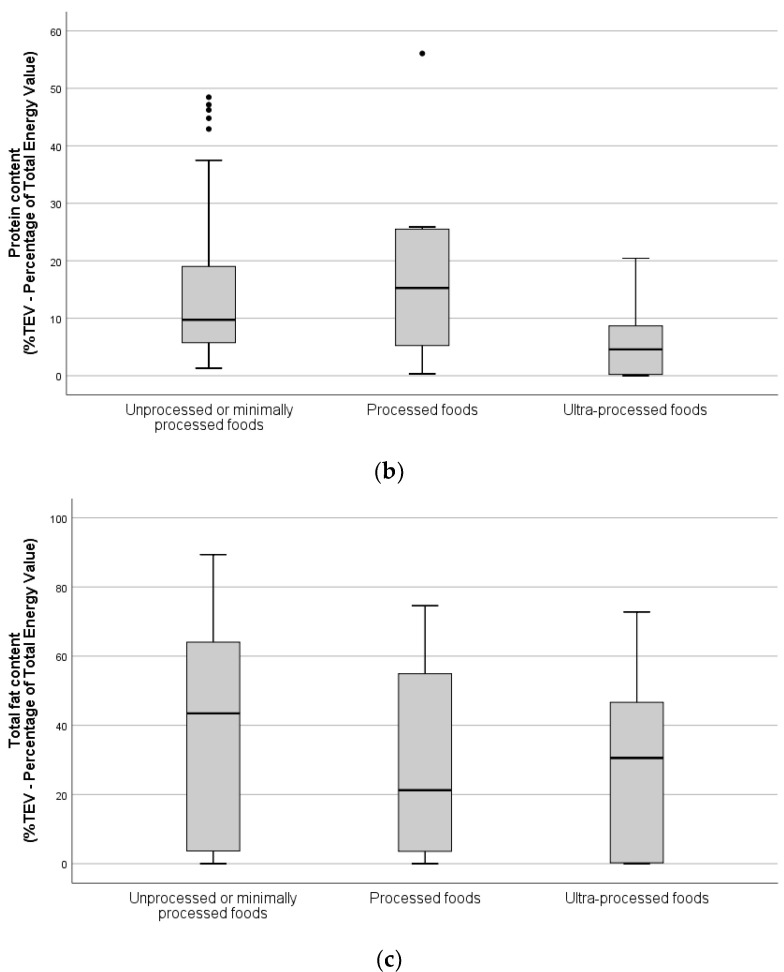
Estimated proportion of total energy value (%TEV) of carbohydrates (**a**), protein (**b**) and total fat (**c**) of the unprocessed/minimally processed, processed, and ultra-processed foods available (*n* = 810, vending sites available for interview).

Comparison of energy content and macronutrient distribution of the three food groups according to degree of processing are presented in Figure 1 and Figure 2, respectively. Energy density of unprocessed or minimally processed foods was significantly lower than processed foods (156 vs. 237 kcal/100 g, *p* = 0.044) and ultra-processed foods (156 vs. 281 kcal/100 g, *p* = 0.003). No significant differences were found regarding energy content per serving. The contribution of protein to TEV in ultra-processed foods was significantly lower than in processed foods (6.8% vs. 14.3%, *p* = 0.041) and unprocessed/minimally processed foods (6.8% vs. 18.6%, *p* = 0.001). No significant differences regarding carbohydrates and fats were observed.

## 4. Discussion

The present study presents relevant data on the nutritional composition and processing degree of the street foods available in Maputo, Mozambique. There was an abundant offer of street food in this setting, as shown by the several hundreds of street food vending sites in only one of its districts. The coexistence of highly processed industrialised food products with natural foods and homemade dishes reflects the nutrition transition phenomenon that Mozambique is currently undergoing. 

Considering the nutrition transition model proposed by Popkin [1], in which this phenomenon is divided into five stages, evidence suggests that Mozambique is passing from stage 3 (Receding famine) to stage 4 (Degenerative disease) [3], and food processing is considered to be one of the main aspects of this phenomenon. Stage 3 is characterised by an increasing consumption of animal sources of protein, vegetables and fruits, as well as a reduction in the intake of starchy staples, in a context of urban growth and increasing income and life expectancy. On the contrary, stage 4 is characterised by a raising availability and consumption of processed foods and beverages rich in sugar, salt, and fat, due to the development of food-transforming technology. Our results regarding the processing degree of the street food offered in Maputo seem to be consistent with the Mozambican position regarding this nutrition transition model. From a morbidity point of view, this represents a shift away from the predominance of communicable diseases and maternal and child nutrition deficiencies (stage 3), towards an increasing prevalence of diet-related NCD (stage 4) [1].

Regarding the processing degree of the street foods available, unprocessed or minimally processed foods (including culinary preparations using them as base) were the most frequently available. Within this group, homemade cooked foods prevailed in both stationary and mobile vendors. Although these homemade dishes were classified within the lowest degree of processing, they might also contain processed ingredients in their composition. A previously published work by Sousa et al. 2018, reporting on analysis of photographic records of the street food vending sites in KaMpfumu, Maputo, showed that processed ingredients, mainly chicken powdered stocks, composed mostly of sodium, were often available for use in culinary preparations [33], and that the homemade dishes most frequently available as street food had concerning values of sodium to potassium ratio, ranging from 1.89 in fried cakes to 11.95 in stewed liver dishes [22]. Findings from this setting also showed that, although traditional culinary practices were still well rooted [8], high consumption of processed ingredients was observed [8,9]. This highlights the importance of implementing public health policies and awareness programs towards the reduction in the use of processed ingredients, not only by street food vendors but also by the general population.

The second most frequently available food products fall within the category of ultra-processed foods. Similar results have been reported by recent studies from the WHO in cities of Central Asia and Eastern Europe, where it was observed a high availability of highly industrialized food products, namely soft drinks, confectionery, fried snacks, and pastries [34,35,36,37,38]. Studies using the food classification based on the extent of food processing showed that the high availability and consumption of ultra-processed food was associated with a lower dietary quality, with higher intakes of energy, saturated fat, added sugar and sodium, as well as lower intakes of fibre, thus having a likely harmful effect on human health [39,40]. Frequent consumption of foods from this group have also been linked to higher body mass index values and greater odds of being overweight or obese, in both adolescents and adults [41]. Considering the potential harmful effects of ultra-processed foods, research on its availability and consumption is of great interest, in the context of prevention of non-communicable diet-related morbidities.

Energy density (kcal/100 g) was significantly higher for ultra-processed foods when compared to unprocessed/minimally processed foods, which was in line with what was found in other settings [35,36]. Although this difference was expected, it was not observed when we compared energy content per serving. These results might be directly explained by the variations on the portion sizes sold. Furthermore, some beverages are classified as ultra-processed, namely alcoholic beverages and soft drinks, which may also contribute to lower the mean energy value per serving of the whole food group. Furthermore, beverages and solid foods do not act as interchangeable items since they are usually consumed together and for that reason their nutritional content should not be compared. Foods included in the unprocessed/minimally processed food group were mostly main courses, which usually presented large serving sizes. On the contrary, ultra-processed foods were most commonly individually packaged, which helps to explain the smaller portions. However, fast food dishes (which are ultra-processed foods, but usually consumed as main courses) showed higher values of energy per serving than most homemade dishes.

The macronutrient profile of the foods assessed in this study showed a widespread predominance, in terms of contribution to energy, of carbohydrates and fats over proteins, as documented in other African settings, namely Benin and Uganda [42,43]. A systematic review on nutritional contribution of street food in developing countries showed that street food presented a high contribution to the daily intakes of fat, trans fat, and sugar [18]. Another work in India showed that snacks sold by street vendors had high contents of total fat, as well as saturated and trans fatty acids [44]. This is of great concern considering the role of those nutrients in the development of obesity and other NCD [45,46,47,48]. The energy and macronutrient contents of the foods and beverages available was widely variable. This was a reflection of the highly heterogeneous street food environment found, including food products with a different number and type of ingredients and degrees of processing, as well as methods of preparation and/or cooking of varying nature and complexity [49].

The main limitation of this study was that the estimation of nutritional composition does not allow us to obtain reliable values of fatty acids within total fat, as well as sugars within carbohydrates, which would be important to understand the nutritional quality of the street foods available. Data from chemical analysis of street foods collected from this setting were recently published [22]. However, it refers only to homemade foods, which are almost all classified as unprocessed or minimally processed foods, whereas, in the present work, the nutritional value was estimated for all the foods and beverages available, both homemade and industrially-produced, thus allowing the comparison between the various groups of processing extent. Detailed nutritional data on street foods with different degrees of processing would be an important recommendation for future work. Finally, although these results are of great relevance to the urban food environment of this setting, their generalizability is limited due to local cultural specificities.

The relationship between the consumption of industrialised foods and beverages and the current pandemics of obesity and NCD is widely accepted [2,50]. In fact, it is currently recognised by the scientific community the importance of considering the extent and purpose of food processing in the context of nutritional and epidemiological studies, in order to strengthen evidence linking food and health [23]. Nonetheless, most of the research addressing dietary changes on developing countries under nutrition transition tend to examine energy and macronutrient intake and/or food groups consumption, frequently not including processed foods as a target [23,39]. Furthermore, evidence suggests that consumption of processed foods will continue to increase in developing countries, if effective measures do not take place [51]. As such, estimating the processing degree of the foods and beverages available for consumption in these countries can be the first step towards the identification of targets for policy intervention.

## 5. Conclusions

In conclusion, street food availability in the district of KaMpfumu, Maputo was high, with unprocessed or minimally processed foods being the most commonly available, followed by ultra-processed foods. In all groups of food processing, carbohydrates were the major contributors to total energy content of the street food available, followed by fats, whereas proteins presented a minor energy contribution. Unprocessed or minimally processed foods presented the lowest energy density, and ultra-processed foods the lowest protein content, when compared to the other food groups. Mobile vending sites sold mostly unprocessed/minimally processed foods, whereas processed and ultra-processed foods were mostly found in stationary vending sites. To our knowledge, this is the first study to provide a characterisation of the street food offered in Mozambique taking into account its extent of processing and nutritional value. Since street food seems to present a significant contribution to the daily intakes of many urban dwellers in this setting, our findings highlight the importance of public health action towards the improvement in the foods and beverages available for consumption on the streets. Integrated information regarding both the nutritional value and processing degree of the foods and beverages being sold should be taken into account when developing and implementing public health strategies targeted at diet-related NCD prevention in this setting. Examples of these strategies may include pricing policies, such as financial support to sellers who use healthier and less processed ingredients, or the application of taxes to ultra-processed foods with lower nutritional density; incentives to the food industry to product reformulation, in order to improve the quality of the processed products available; and nutritional education, to raise awareness of both street food vendors and consumers regarding the health implications of the frequent consumption of highly-processed food products, as well as the frequent inclusion of these foods in culinary preparations. In terms of future research, chemical analyses of the most frequently available foods from all the processing extent groups (including, for example, information on fatty acids, simple sugars, and some micronutrients) would be important in providing more detailed data regarding their nutritional value considering processing degree. The improvement of nutritional data available, especially in food labels (namely on fatty acids, sugar, and sodium contents), would also be an important progress, not only for their use in research, but also towards adequate nutrition information provided to consumers.

## Figures and Tables

**Table 1:** Ready-to-eat foods and beverages available in mobile and stationary street food vending sites in KaMpfumu, Maputo, Mozambique, according to degree of processing (*n* = 810, available for interview).

	N ^a^	Total *n* = 810	Mobile SF Vending Sites *n* = 187	Stationary SF Vending Sites *n* = 623
		*n* (%)	*n* (%)	*n* (%)
Unprocessed or minimally processed foods (including culinary preparations using them as base) ^b^	73	571 (70.5)	161 (86.1)	410 (65.8)
Rice Corn ^c^ Wheat ^d^ Beef ^e^ Pork ^e^ Poultry ^e^ Goat ^e^ Liver ^e^ Fish/seafood ^f^ Eggs ^e^ Milk ^g^ Beans ^h^ Nuts ^i^ Cassava ^j^ Vegetables (except leaves) ^k^ Leaves ^l^ Potatoes Fruit Water and tea	1 4 5 2 2 4 1 1 5 2 3 2 4 2 2 4 2 25 2	47 (8.2) 52 (9.1) 75 (13.1) 46 (8.0) 15 (2.6) 56 (9.8) 9 (1.6) 2 (0.3) 69 (12.0) 88 (15.4) 17 (3.0) 102 (17.8) 62 (10.8) 7 (1.2) 25 (4.4) 16 (2.8) 63 (11.0) 204 (35.7) 172 (30.0)	0 (0.0) 0 (0.0) 25 (15.5) 0 (0.0) 0 (0.0) 0 (0.0) 0 (0.0) 0 (0.0) 3 (1.9) 34 (21.1) 0 (0.0) 24 (14.9) 4 (2.5) 5 (3.1) 0 (0.0) 0 (0.0) 2 (1.2) 70 (43.5) 1 (0.6)	47 (11.5) 52 (12.7) 50 (12.2) 46 (11.2) 15 (3.7) 56 (13.7) 9 (2.2) 2 (0.5) 66 (16.1) 54 (13.2) 17 (4.1) 78 (19.0) 58 (14.1) 2 (0.5) 25 (6.1) 16 (3.9) 61 (14.9) 134 (32.7) 171 (41.7)
Processed foods ^b^	8	119 (14.7)	16 (8.6)	103 (16.5)
Sandwiches Bread Canned fish Ham Cheese Fermented alcoholic beverages	1 2 1 1 1 2	58 (48.7) 81 (68.1) 3 (2.5) 1 (0.8) 11 (9.2) 28 (23.5)	11 (68.8) 16 (100.0) 0 (0.0) 0 (0.0) 0 (0.0) 0 (0.0)	47 (45.6) 65 (63.1) 3 (2.9) 1 (1.0) 11 (10.7) 28 (27.2)
Ultra-processed foods ^b^	27	478 (59.0)	32 (17.1)	446 (71.6)
Bun Fast food dishes ^m^ Cakes and cookies Fried snacks ^n^ Confectionery ^o^ Industrial popcorn Sausages Soft drinks Sugar-free soft drinks Distilled alcoholic beverages Milk products ^p^	1 4 3 2 4 1 2 4 2 2 2	4 (0.8) 30 (6.3) 302 (63.0) 198 (41.3) 274 (57.2) 3 (0.6) 48 (10.0) 252 (52.6) 11 (2.3) 14 (2.9) 21 (4.4)	2 (6.3) 0 (0.0) 3 (9.4) 5 (15.6) 10 (31.3) 1 (3.1) 12 (37.5) 2 (6.3) 0 (0.0) 0 (0.0) 1 (3.1)	2 (0.4) 30 (6.7) 299 (66.9) 193 (43.2) 264 (59.1) 2 (0.4) 36 (8.1) 250 (55.9) 11 (2.5) 14 (3.1) 20 (4.5)

SF, street food. ^a^ Number of different foods that were included in each food category/group; ^b^ The sum of all food categories may be higher than the total N of the respective food group, because the same SF vending site can have more than one food available; ^c^ Includes *massaroca* (grilled corn cob), *xima* (side dish made with corn flour and water), and homemade popcorn; ^d^ Includes homemade wheat-based cakes and biscuits, as well as pasta dishes; ^e^ Includes different culinary preparations (e.g., stewed, fried); ^f^ Includes fried fish, stewed squid, and fish samosas; ^g^ Includes milk, tea with milk and reconstituted powdered milk; ^h^ Includes *badjias* (fried dumplings made with beans or bean flour, with onion and garlic) and stewed beans; ^i^ Includes peanut curry dishes, peanuts and cashews; ^j^ Includes cassava (raw) and *molina*/*lifete* (homemade sweet made with cassava flour as a base ingredient, with roasted peanuts and sugar); ^k^ Includes salad (lettuce and tomato) and vegetables soup; ^l^ Includes *matapa*, *m’boa*, and *cacana* (main dishes made with leaves as a base ingredient, with coconut and peanuts); ^m^ Includes industrial pizza, hamburgers, and hot-dogs; ^n^ Includes pre-fried samosas and chips; ^o^ Includes industrial ice-cream, chocolates, and other candies; ^p^ Includes milk-based industrial drinks and sweetened or flavoured yogurt.

**Table 2:** Estimated energy content and macronutrient distribution of the ready-to-eat food products available from street food vending sites in KaMpfumu, Maputo, Mozambique, according to degree of processing (*n* = 810, available for interview).

	n ^a^	Serving Size (g)	Energy (kcal)		Contribution to TEV (%)		
			/Serving	/100 g	Carbohydrates	Protein	Fats
		Mean (Min–Max)	Mean (Min–Max)	Mean (Min–Max)	Mean (Min–Max)	Mean (Min–Max)	Mean (Min–Max)
Unprocessed or minimally processed foods (including culinary preparations using them as base) ^b^	73	212 (11–679)	278 (0–1305)	156 (0–589)	49.5 (2.5–100)	14.3 (1.3–48.5)	36.0 (0.0–89.4)
Rice	1	141	476	338	74.2	5.7	18.3
Corn ^c^	4	248 (75–530)	243 (111–341)	176 (61–454)	79.0 (64.5–90.7)	7.5 (3.5–13.0)	12.1 (2.6–31.4)
Wheat ^d^	5	108 (11–262)	251 (49–427)	319 (163–438)	51.3 (38.7–69.8)	8.1 (4.2–11.6)	38.9 (18.3–55.7)
Beef ^e^	2	220 (169–271)	394 (341–446)	184 (165–203)	5.7 (4.1–7.3)	27.1 (24.8–29.4)	66.8 (66.4–67.3)
Pork ^e^	2	226 (182–271)	408 (360–455)	183 (168–198)	3.3 (2.5–4.1)	32.0 (28.5–35.6)	65.2 (62.7–67.8)
Poultry ^e^	4	429 (273–679)	479 (438–511)	127 (75–187)	8.0 (2.6–12.8)	39.3 (29.6–47.2)	52.1 (38.7–65.6)
Goat ^e^	1	291	303	104	6.8	46.2	46.3
Liver ^e^	1	217	359	165	7.5	33.2	58.7
Fish/seafood ^f^	5	219 (59–528)	251 (129–444)	164 (78–265)	14.8 (7.4–25.0)	30.7 (11.6–48.5)	53.6 (43.4–66.0)
Eggs ^e^	2	220 (56–383)	279 (93–465)	143 (121–164)	7.2 (2.9–11.5)	24.7 (19.4–30.0)	67.9 (67.7–68.2)
Milk ^g^	3	228 (200–283)	119 (62–170)	51 (31–62)	35.9 (30.3–46.9)	19.1 (16.1–20.7)	45.1 (36.6–49.4)
Beans ^h^	2	129 (29–229)	238 (80–395)	226 (173–280)	17.9 (7.3–28.5)	12.9 (2.3–23.5)	67.5 (45.7–89.4)
Nuts ^i^	4	224 (38–657)	556 (127–926)	411 (141–589)	20.1 (6.1–44.9)	14.5 (9.4–18.6)	64.5 (44.2–75.3)
Cassava ^j^	2	100	312 (153–471)	312 (153–471)	71.0 (45.3–96.7)	5.8 (1.8–9.7)	22.4 (1.2–43.5)
Vegetables (except leaves) ^k^	2	382 (124–641)	111 (64–158)	38 (25–51)	40.3 (26.3–54.2)	10.3 (8.4–12.2)	45.6 (30.0–61.2)
Leaves ^l^	4	371 (300–438)	977 (838–1306)	263 (235–298)	10.9 (8.5–12.4)	13.3 (12.5–14.9)	72.7 (69.9–76.1)
Potatoes	2	289 (280–299)	305 (218–392)	105 (78–131)	65.0 (49–81)	7.2 (4.6–9.7)	22.3 (1.2–43.4)
Fruit	25	155 (60–500)	128 (25–964)	78 (13–375)	83.8 (11.7–100.0)	5.7 (1.3–12.0)	12.4 (0.0–84.0)
Water and tea	2	200	0	0	-	-	-
Processed foods ^b^	8	85 (10–165)	170 (21–386)	237 (85–400)	33.1 (0.2–80.6)	18.6 (0.3–56.1)	29.3 (0.0–74.6)
Sandwiches	1	165	336	204	59.2	18.4	20.0
Bread	2	55 (10–100)	147 (40–253)	327 (253–400)	75.3 (70.0–80.6)	11.1 (10.0–12.1)	14.8 (7.1–22.5)
Canned fish	1	125	386	309	1.3	25.9	72.8
Ham	1	20	21	107	6.7	56.1	37.0
Cheese	1	10	37	374	0.2	25.1	74.6
Fermented alcoholic beverages	2	125 (100–150)	142 (124–160)	125 (85–165)	23.6 (13.0–34.2)	0.4 (0.3–0.5)	0.0 (0.0)
Ultra-processed foods ^b^	27	151 (15–375)	292 (1–873)	281 (0–610)	51.2 (0.0–100.0)	6.8 (0.0–22.2)	29.7 (0.0–72.8)
Bun	1	50	171	341	63.3	11.7	23.8
Fast food dishes ^m^	4	239 (158–375)	546 (282–784)	234 (179–298)	33.6 (22.4–42.0)	17.1 (14.0–19.9)	47.2 (36.2–57.1)
Cakes and cookies	3	110 (40–189)	513 (177–873)	466 (442–491)	53.0 (46.9–62.5)	5.3 (4.6–5.9)	40.8 (30.6–47.9)
Fried snacks ^n^	2	100 (40–160)	454 (72–836)	351 (180–521)	50.9 (40.6–61.1)	3.9 (3.3–4.6)	43.3 (32.5–54.1)
Confectionery ^o^	4	77 (43–100)	326 (160–520)	419 (236–527)	57.4 (44.3–86.6)	3.6 (1.2–5.5)	38.8 (12.2–49.9)
Industrial popcorn	1	100	522	522	48.3	4.3	44.8
Sausages	2	28 (15–40)	93 (92–94)	423 (235–610)	7.1 (6.8–7.3)	21.3 (20.4–22.2)	68.1 (63.4–72.8)
Soft drinks	4	245 (200–330)	140 (113–198)	61 (41–99)	98.1 (94.5–100.0)	0.1 (0.0–0.5)	0.1 (0.0–0.5)
Sugar-free soft drinks	2	290 (250–330)	5 (1–10)	2 (0–4)	100.0	0.0	0.0
Milk products ^p^	2	225 (200–250)	151 (65–238)	64 (33–95)	70.3 (68.9–71.6)	8.7 (8.5–8.8)	21.0 (20.0–22.2)
Distilled alcoholic beverages	2	40	103 (100–105)	257 (250–263)	0.1 (0.0–0.2)	0.0	0.0

SF, street food; TEV, total energy value. ^a^ Number of different foods that were included in each food category/group; ^b^ The sum of all food categories may be higher than the total N of the respective food group, because the same SF vending site can have more than one food available; ^c^ Includes *massaroca* (grilled corn cob), *xima* (side dish made with corn flour and water) and homemade popcorn; ^d^ Includes homemade wheat-based cakes and biscuits, as well as pasta dishes; ^e^ Includes different culinary preparations (e.g., stewed, fried); ^f^ Includes fried fish, stewed squid and fish samosas; ^g^ Includes milk, tea with milk and reconstituted powdered milk; ^h^ Includes *badjias* (fried dumplings made with beans or bean flour, with onion and garlic) and stewed beans; ^i^ Includes peanut curry dishes, peanuts, and cashews; ^j^ Includes cassava (raw) and *molina*/*lifete* (homemade sweet made with cassava flour as a base ingredient, with roasted peanuts and sugar); ^k^ Includes salad (lettuce and tomato) and vegetables soup; ^l^ Includes *matapa*, *m’boa*, and *cacana* (main dishes made with leaves as a base ingredient, with coconut and peanuts); ^m^ Includes industrial pizza, hamburgers, and hot-dogs; ^n^ Includes pre-fried samosas and chips; ^o^ Includes industrial ice-cream, chocolates, and other candies; ^p^ Includes milk-based industrial drinks and sweetened or flavoured yogurt.

## Data Availability

The data presented in this study are available upon request to the corresponding author.

## References

[1] Popkin B.M. (2006). Global nutrition dynamics: The world is shifting rapidly toward a diet linked with noncommunicable diseases. Am. J. Clin. Nutr..

[2] Baker P., Friel S. (2014). Processed foods and the nutrition transition: Evidence from Asia. Obes. Rev..

[3] Nnyepi M.S., Gwisai N., Lekgoa M., Seru T. (2015). Evidence of nutrition transition in Southern Africa. Proc. Nutr. Soc..

[4] Popkin B.M. (2011). Contemporary nutritional transition: Determinants of diet and its impact on body composition. Proc. Nutr. Soc..

[5] Vorster H.H., Kruger A., Margetts B.M. (2011). The nutrition transition in Africa: Can it be steered into a more positive direction?. Nutrients.

[6] Hawkes C. (2006). Uneven dietary development: Linking the policies and processes of globalization with the nutrition transition, obesity and diet-related chronic diseases. Glob. Health.

[7] Padrao P., Laszczynska O., Silva-Matos C., Damasceno A., Lunet N. (2012). Low fruit and vegetable consumption in Mozambique: Results from a WHO STEPwise approach to chronic disease risk factor surveillance. Br. J. Nutr..

[8] Silva V., Santos S., Novela C., Padrão P., Moreira P., Lunet N. Some observations on food consumption and culinary practices in Maputo, Mozambique. Proceedings of the Culinary Arts and Sciences VIII: Global, Local, and National Perspectives.

[9] Smart J.C., Tschirley D., Smart F. (2020). Diet Quality and Urbanization in Mozambique. Food Nutr. Bull..

[10] Institute for Health Metrics and Evaluation Institute for Health Metrics and Evaluation. Global Burden of Diseases (GBD) Compare. http://vizhub.healthdata.org/gbd-compare/.

[11] Fontes F., Damasceno A., Jessen N., Prista A., Silva-Matos C., Padrão P., Lunet N. (2019). Prevalence of overweight and obesity in Mozambique in 2005 and 2015. Public Health Nutr..

[12] Global Nutrition Report. Country Nutrition Profiles: Mozambique. https://globalnutritionreport.org/resources/nutrition-profiles/africa/eastern-africa/mozambique/.

[13] Food and Agriculture Organization (2011). Nutrition Country Profile—Republic of Mozambique.

[14] Popkin B.M. (1999). Urbanization, Lifestyle Changes and the Nutrition Transition. World Dev..

[15] Winarno F., Allain A. (1991). Street Foods in Developing Countries: Lessons from Asia.

[16] Fellows P., Hilmi M. (2012). Selling Street and Snack Foods.

[17] Liu Z., Zhang G., Zhang X. (2014). Urban street foods in Shijiazhuang city, China: Current status, safety practices and risk mitigating strategies. Food Control.

[18] Steyn N.P., McHiza Z., Hill J., Davids Y.D., Venter I., Hinrichsen E., Opperman M., Rumbelow J., Jacobs P. (2014). Nutritional contribution of street foods to the diet of people in developing countries: A systematic review. Public Health Nutr..

[19] World Health Organization (2017). FEEDcities Project: The Food Environment Description in Cities in Eastern Europe and Central Asia—Tajikistan.

[20] Raimundo I., Crush J., Pendleton W. (2014). The State of Food Insecurity in Maputo, Mozambique.

[21] Salamandane A., Silva A.C., Brito L., Malfeito-Ferreira M. (2021). Microbiological assessment of street foods at the point of sale in Maputo (Mozambique). Food Qual. Saf..

[22] Sousa S., Gelormini M., Damasceno A., Lopes S.A., Malo S., Chongole C., Muholove P., Casal S., Pinho O., Moreira P. (2019). Street food in Maputo, Mozambique: Availability and nutritional value of homemade foods. Nutr. Health.

[23] Fardet A., Rock E., Bassama J., Bohuon P., Prabhasankar P., Monteiro C., Moubarac J.C., Achir N. (2015). Current food classifications in epidemiological studies do not enable solid nutritional recommendations for preventing diet-related chronic diseases: The impact of food processing. Adv. Nutr..

[24] Monteiro C.A., Cannon G., Levy R.B., Moubarac J.C., Jaime P., Martins A.P., Canella D., Louzada M., Parra D., Ricardo C. (2016). Food classification. Public health: NOVA. The star shines bright. J. World Public Health Nutr. Assoc..

[25] Gelormini M., Damasceno A., Lopes S.A., Malo S., Chongole C., Muholove P., Casal S., Pinho O., Moreira P., Padrao P. (2015). Street Food Environment in Maputo (STOOD Map): A Cross-Sectional Study in Mozambique. JMIR Res. Protoc..

[26] Food and Agriculture Organization (1989). Food and Nutrition Paper No. 46: Street Foods.

[27] World Health Organization (1996). Food Safety Team. Essential Safety Requirements for Street-Vended Foods.

[28] AURECON (2012). Comprehensive Urban Transport Master Plan for the Greater Maputo Region—Survey Package A. Maputo: Inventory Surveys. Final Report.

[29] Conselho Municipal de Maputo (2010). Perfil Estatístico do Município de Maputo.

[30] Direcção Nacional de Saúde (1991). Tabela de Composição de Alimentos.

[31] Núcleo de Estudos e Pesquisas em Alimentação (2011). Tabela Brasileira de Composição de Alimentos.

[32] SAFOODS South African Food Data System. http://safoods-apps.mrc.ac.za/foodcomposition/.

[33] Sousa S., Damasceno A., Gelormini M., Jessen N., Lunet N., Padrao P. (2017). Powdered chicken stock may be an important source of dietary sodium intake in Maputo, Mozambique. J. Public Health.

[34] Albuquerque G., Gelormini M., de Morais I.L., Sousa S., Casal S., Pinho O., Moreira P., Breda J., Lunet N., Padrão P. (2020). Street food in Eastern Europe: A perspective from an urban environment in Moldova. Br. J. Nutr..

[35] Albuquerque G., Lança de Morais I., Gelormini M., Sousa S., Casal S., Pinho O., Moreira P., Breda J., Lunet N., Padrão P. (2020). Macronutrient composition of street food in Central Asia: Bishkek, Kyrgyzstan. Food Sci. Nutr..

[36] Albuquerque G., Morais I., Gelormini M., Casal S., Damasceno A., Pinho O., Moreira P., Jewell J., Breda J., Lunet N. (2019). Street food in Dushanbe, Tajikistan: Availability and nutritional value. Br. J. Nutr..

[37] World Health Organization (2019). FEEDcities Project: Food Environment Description in Cities—Eastern Europe and Central Asia. Banja Luka. The Republika Srpska. Bosnia and Herzegovina.

[38] World Health Organization (2019). FEEDcities Project: Food Environment Description in Cities—Eastern Europe and Central Asia. Sarajevo. The Federation of Bosnia and Herzegovina. Bosnia and Herzegovina.

[39] Monteiro C.A., Levy R.B., Claro R.M., de Castro I.R., Cannon G. (2011). Increasing consumption of ultra-processed foods and likely impact on human health: Evidence from Brazil. Public Health Nutr..

[40] Moubarac J.C., Martins A.P., Claro R.M., Levy R.B., Cannon G., Monteiro C.A. (2013). Consumption of ultra-processed foods and likely impact on human health. Evidence from Canada. Public Health Nutr..

[41] Louzada M.L., Baraldi L.G., Steele E.M., Martins A.P., Canella D.S., Moubarac J.C., Levy R.B., Cannon G., Afshin A., Imamura F. (2015). Consumption of ultra-processed foods and obesity in Brazilian adolescents and adults. Prev. Med..

[42] Nago E.S., Lachat C.K., Huybregts L., Roberfroid D., Dossa R.A., Kolsteren P.W. (2010). Food, energy and macronutrient contribution of out-of-home foods in school-going adolescents in Cotonou, Benin. Br. J. Nutr..

[43] Namugumya B.S., Muyanja C. (2012). Contribution of street foods to the dietary needs of street food vendors in Kampala, Jinja and Masaka districts, Uganda. Public Health Nutr..

[44] Gupta V., Downs S.M., Ghosh-Jerath S., Lock K., Singh A. (2016). Unhealthy Fat in Street and Snack Foods in Low-Socioeconomic Settings in India: A Case Study of the Food Environments of Rural Villages and an Urban Slum. J. Nutr. Educ. Behav..

[45] European Union (2014). Workshop: Trans Fats.

[46] Gulati S., Misra A. (2014). Sugar intake, obesity, and diabetes in India. Nutrients.

[47] Remig V., Franklin B., Margolis S., Kostas G., Nece T., Street J.C. (2010). Trans fats in America: A review of their use, consumption, health implications, and regulation. J. Am. Diet. Assoc..

[48] World Health Organization (2015). Information Note about Intake of Sugars Recommended in the WHO Guideline for Adults and Children.

[49] Draper A. (1996). Street foods in developing countries: The potential for micronutrient fortification.

[50] World Health Organization (2003). Diet, Nutrition and the Prevention of Chronic Diseases. Report of a Joint WHO/FAO Expert Consultation. WHO Technical Report Series No. 916.

[51] Monteiro C.A., Moubarac J.C., Cannon G., Ng S.W., Popkin B. (2013). Ultra-processed products are becoming dominant in the global food system. Obesity Rev..

